# Influence of substrate curvature on osteoblast orientation and extracellular matrix deposition

**DOI:** 10.1186/1754-1611-7-23

**Published:** 2013-10-03

**Authors:** Marcello Pilia, Teja Guda, Stefanie M Shiels, Mark R Appleford

**Affiliations:** 1 Department of Biomedical Engineering, University of Texas, San Antonio, TX, USA; 2 Joint Program in Biomedical Engineering, University of Texas Health Science Center at San Antonio/University of Texas, San Antonio, TX, USA

**Keywords:** Hydroxyapatite, Osteon architecture, Curvature, Microchannels, Extracellular Matrix, Nano-indentation

## Abstract

**Background:**

The effects of microchannel diameter in hydroxyapatite (HAp) substrates on osteoblast behavior were investigated in this study. Microchannels of 100, 250 and 500 μm diameter were created on hydroxyapatite disks. The changes in osteoblast precursor growth, differentiation, extra cellular matrix (ECM) secretion and cell attachment/orientation were investigated as a function of microchannel diameter.

**Results:**

Curvature did not impact cellular differentiation, however organized cellular orientation was achieved within the 100 and 250 μm microchannels (mc) after 6 days compared to the 12 days it took for the 500mc group, while the flat substrate remained disorganized. Moreover, the 100, 250 and 500mc groups expressed a specific shift in orientation of 17.45°, 9.05°, and 22.86° respectively in 24 days. The secreted/mineralized ECM showed the 100 and 250mc groups to have higher modulus *(E)* and hardness (*h*) (E = 42.6GPa; h = 1.6GPa) than human bone (E = 13.4-25.7GPa; h = 0.47-0.74GPa), which was significantly greater than the 500mc and control groups (p < 0.05). It was determined that substrate curvature affects the cell orientation, the time required for initial response, and the shift in orientation with time.

**Conclusions:**

These findings demonstrate the ability of osteoblasts to organize and mineralize differentially in microchannels similar to those found in the osteons of compact bone. These investigations could lead to the development of osteon-like scaffolds to support the regeneration of organized bone.

## Introduction

Natural bone achieves much of its mechanical strength through cortical bone, specifically through the organization of its osteons. The structural organization of native bone directly contributes to the mechanical strength of bone tissue, which is critical since load bearing and providing mechanical support are the primary functions of the skeleton. The lamellar rings that surround the central microchannel-like structure of the osteon are formed by the secretion of Type-I Collagen (Col-I) by osteoblasts during osteonal development. While Col-I and other organic molecules make up 70% of total bone composition, the remaining 30% is inorganic, composed of bone minerals, specifically nano-size crystals of hydroxyapatite (HAp) [1]. The bone minerals are responsible for the hardness of bone whereas the organic portion gives skeletal tissue its elasticity [2]. In addition, the secretion of Col-I along different orientations, followed by the deposition of bone minerals, gives cortical bone its high compressive strength and toughness [3]. The compressive strength of cortical bone ranges between 100–230 MPa, whereas trabecular bone ranges between 2–12 MPa [4].

**Figure 1:**
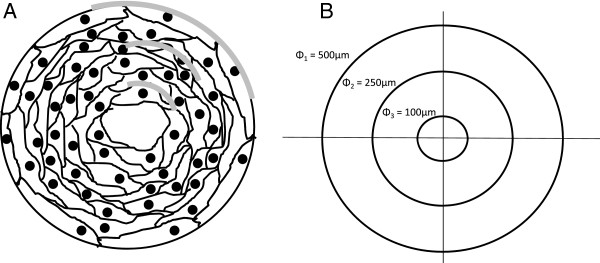
**Drawing (A) and representation (B) of the concentric ring structure typical of the osteon. A)** The osteonal concentric ring structure ranges from 50 to 500 μm in diameter. As multiple layers of cells are deposited towards the middle of the osteon the osteocytes are exposed to different substrate curvature (gray line). This was simulated by fabricating microchannels of various diameters where HFOb were seeded for up to 24 days. **B)** a graphical representation of the different curvatures the cells are exposed to in all the different substrates. The smaller the diameter of the microchannel, the more curved the substrate becomes.

Regeneration of bony defects is a growing problem, as bone fracture and trauma related injuries account for more than 1.3 million procedures in the United States [5,6]. Specifically, critical sized segmental bone defects (SBDs) are a cause of significant concern in orthopedics because they do not heal on their own [7-12]. Autografts are the gold standard approach favored by surgeons to treat SBDs [7,8,10,12,13]. Although this technique is widely used, it is also associated with various drawbacks, including infections and donor site morbidity [8]. In recent years, the advent of bone tissue engineering has brought new ideas, discovery, development and characterization of new biomaterials for regenerative purposes. Tissue engineered grafts for SBDs should promote vascularity through highly interconnected pores [14] while maintaining the structural and mechanical integrity similar to native tissue [15]. Current bone tissue engineering approaches include cell-based, scaffold-based, and delivery-based strategies [10]. Even though in recent times there has been an increase in scientific publications aimed at increasing the mechanical properties of biomaterials [16,17] and underlining the importance of mechanotransduction [18-20], mechanical stability remains a weak component of these approaches. Current tissue engineered scaffolds can only match the strength range of trabecular bone [21] making the implants unsuitable for weight-bearing applications without secondary fixation. Transitory metallic plates have become a necessity to be placed in conjunction with the SBD scaffolds [22,23] to divert the load away from the fragile scaffold. A drawback of these plates is stress shielding effects around the defect area, slowing down the healing response and limiting healthy bone regeneration at the scaffold [24]. The two factors that have received the most attention in bone tissue engineering to overcome these short-comings are material composition and architecture of the scaffolds [25]. Various types of degradable scaffolds have been investigated and developed over the years for bone repair using ceramics, polymers, and combinations of the two material types [21]. Synthetic HAp, has been used as a material for bone tissue engineering because of its biocompatibility, bioactivity [26-28] and its compositional resemblance to the inorganic phase of natural bone [29]. Current investigations waiver toward HAp scaffolds that are highly porous and have architectures resembling that of trabecular bone [23]. In contrast, HAp scaffolds composed of microchannel-like structures resembling the architecture of cortical bone remains virtually untested in the literature. When comparing osteoblast attachment and proliferation, the geometry of the substrate to which the cell attaches differs between the filament-like, convex surface of the trabeculae and the tubular-like, concave surface of the osteons. Yet, the diameter of the osteon ring to which the cell attaches changes continuously [30]. Black *et al.* reported the average osteon size to range between 50 and 500 μm in diameter [30]. When the osteoblast attaches to the outermost layer of the osteon (~500 μm in diameter) the cell (20-30 μm in size) is exposed to a nearly flat surface [31]. As bone is deposited, the concentric layers stack and the cells begin to attach to an increasingly curved substrate, eventually forming the innermost osteonal layer (~50 μm diameter). A graphical representation to help understand this concept can be seen in Figure 1. This leads to the hypothesis that as osteoblasts are exposed to substrates of varying curvature, the ECM that is secreted is organized variably from layer to layer. It is critical to first gain understanding of how osteoblasts grow and differentiate within the osteon and determine how substrate curvature affects cell responses. Few studies have been conducted to analyze the effect of substrate curvature on cell attachment, orientation and growth. Studies have focused on the strength of attachment of cytoskeletal filaments on spherical and micropatterned substrates [32-38]. Specifically, Noireaux *et al.* studied the growth of an actin gel around spherical beads to determine cell membrane deformation and cell motility direction [36], Schwartz *et al.* investigated the role of substrate curvature in actin pushing forces [38], Pathak *et al.* investigated focal adhesion development on Y, V, T, and U shape micropatterned substrate [37], Lehnert *et al.* and Balaban *et al.* investigated cell behavior on linear and hole-micropatterned substrates [32,34]; Lam *et al.* created wavy micropatterns out of silicone rubber and attached myoblast precursor cells to determine myotube formation [33], Nikkah *et al.* investigated the change in fibroblast behavior when exposed to 3D silicon microstructures [35], and Holthaus *et al.* investigated the effects of microchannel width in osteoblast orientation [16]. However, limited literature is available to show how the change in substrate curvature diameter affects cellular response, bone ECM secretion or bone marker activation.

The goal of this study is to determine the effect of curved substrates on osteoblast growth, differentiation and organization within the microchannels, as well as ECM secretion, mineralization and hardness as a function of the substrate curvature. To accomplish this, HAp substrates with microchannels of various diameters were built to match the microchannel diameter range of natural osteons. On these patterned substrates, an osteoblast precursor cell was cultured *in vitro* to investigate cell responses to the various curvatures.

## Materials and methods

### Mold fabrication

**Figure 2:**
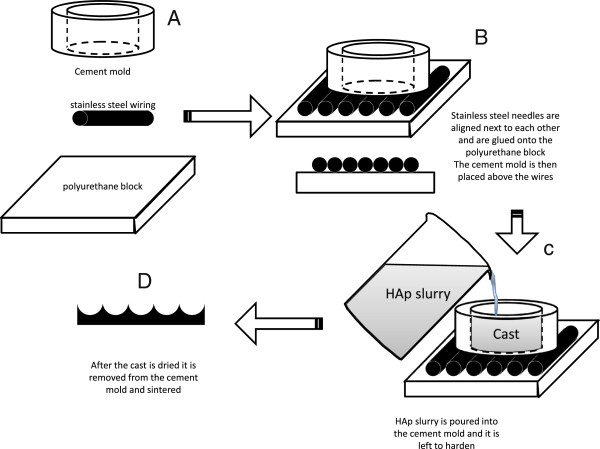
**Diagram showing the steps used to create the molds onto which the HAp was cast. (A)** First stainless steel wires of different dimensions were cut 25 mm long and were lined up next to each other in the axial direction on a polyurethane block to form the negative template. **(B)** These needles were then adhered with epoxy to the PU block to prevent movement. **(C)** The HAp was then cast into a cement mold (10 mm diameter and 6 mm depth) to form the positive cast. The cast HAp disk was then removed and sintered. **(D)** The resulting pattern was a series of concave curves.

Half cylinder molds were fabricated using stainless steel wire (HM Wire International Inc. – Canton, OH) of three different diameters: 110, 320, and 750 μm. The wire was cut in 25 mm sections that were positioned adjacent to each other with no overlap or additional-spacing. Sufficient wires were arranged to form a square 25 × 25 mm. Dental stone cement (Coecal™ Type III Dental Stone – GC America Inc, Alsip, IL) was mixed with distilled water (3:1 w/v ratio) to form molds into which HAp slurry were cast. Each cement mold measured 5 mm in height (Figure 2). Subsequently a hole (ϕ = 10 mm) was drilled in the center for fabrication of the Hap disks. The cement mold was then placed directly above the wire and was held securely in place by vice compression. Details illustrating mold fabrication are found in Figure 2A-C.

### Fabrication of the HAp disks

The HAp disks were made by solution casting as shown in Figure 2D. A HAp slurry was created by using a previously described method [39] using synthetic nano-size HAp (OssGen, South Korea). Briefly, the binders used to stabilize the slurry structure included 3% high molecular weight polyvinyl alcohol, 1% v/v carboxymethylcellulose, 1% v/v ammonium polyacrylate dispersant, and 3% v/v N,N-dimethylformamide drying agent. The solution was then cast into the constructed molds and sintered in a high temperature furnace (Thermolyne, Dubuque, IA). Before sintering, each disk measured 10 mm diameter and 5 mm height. The sintering process profile contained a ramp increase in temperature of 5°C/min up to 300°C with a hold time of 1 hour, then up at the same rate to 600°C for 1 hour hold, and finally to 1230°C for a 5 hour hold. The sintered disks were then cooled at a rate of 5°C/min until room temperature is achieved.

### Characterization of the disks

**Figure 3:**
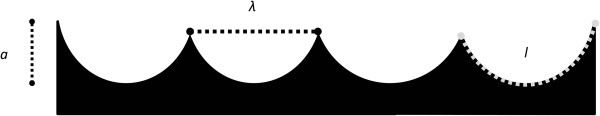
**Graphical representation of the longitudinal section of the newly developed HAp disks.** During characterization the wavelength (*λ*), amplitude (*a*) and the arc length (*l*) were measured from ceramic image analysis.

The resulting disks were analyzed for template geometry and architecture using scanning electron microscopy (SEM) and light microscopy. Laser profilometry (Bruker Contour GT, Tucson, AZ) was used to determine the surface roughness of the disks. First, a disk of each template size was embedded in Pelco® Fast Curing Epoxy Hardener (TedPella Inc, Redding, CA) and a microtome saw (Leica SP1600; Wetzlar, Germany) was used to cut the samples in a direction perpendicular to the template microchannels. Each section cut was roughly 100 μm thick and was polished on both sides using a wet-sander (Struers LaboPol-5, 800 → 1200 grit paper; Cleveland, OH). Each section was analyzed under a microscope (Leica DMIL LED, Wetzlar, Germany), where software (Bioquant Osteo 2010, Nashville, TN) was used to acquire *λ* (wavelength), *a* (amplitude), *l* (arc length), and *d*_*d*_ (disk diameter) (Figure 3). *λ* is the average distance between two peaks; *a* is the distance between the peaks and the valleys; *l* is the length of the arc between two peaks. The surface area (A_e_) was calculated using Equations 1–3, where *n* is the number of channels within each disk and *d*_*e*_ is the diameter of the ellipse. Following characterization, the disks used for cell culture were sterilized by ethylene oxide (AN74i Anprolene gas sterilizer, Andersen Sterilizers, Inc) for a 12 hour cycle.

(1) $$n=\frac{d_{d}}{\lambda }$$

(2) $$d_{e}=n\times l$$

(3) $$A_{e}=\pi \frac{d_{d}}{2}\frac{d_{e}}{2}$$

### Human fetal osteoblast cell culture

Human Fetal Osteoblasts (HFObs) (Cell Applications, Inc.- San Diego, CA) were used to evaluate bone cell response to the varying curvatures. Even though primary Mesenchymal cells are pluripotent compared to HFObs which are committed to the osteogenic lineage, they were not used because the objective of this study was to investigate the effect of local micro-architecture on the matrix production, organization and maturation of osteoblast-like cells, rather than to investigate the commitment of progenitor cells to an osteoblast-like phenotype. The cells were cultured in growth media containing Dubecco Modifed Eagle Medium (DMEM), 10% Fetal Bovine Serum (FBS), and 1% Penicillin Streptomycin Amphotericin B Solution (PSA) (all purchased from Invitrogen, USA). When cells reached confluence on the cell culture-flask, the HFObs were washed with phosphate buffered saline (PBS) and then 0.25% Trypsin/EDTA was added in osteogenic media (DMEM, 3% FBS, 1% PSA, 10 mM Glycerolphosphate, 50 μg/mL Ascorbic acid and 10nM Dexamethasone). The cells in solution were counted (Z_2_ Coulter® Particle Count and Size Analyzer; Beckman Coulter™ - Brea, CA) and seeded on the disks at confluence (55,000 cells/cm^2^). Four time points were tested: 6, 12, 18 and 24 days (n = 12). For n = 8 disks, media was collected. Each disk was then washed with PBS, followed by cell permeabilization using 0.1% Triton X-100 in PBS (PBS-T), and after a freeze/thaw cycle the supernatant was collected. The remaining n = 4 disks were stored in 4% formaldehyde for imaging.

#### *In vitro osteoblast tracking on the HAp disks*

Cell numbers were measured directly from the cell lysate solution. Specifically, 25 μL of lysate was added to the Quant-iT™ PicoGreen® dsDNA kit (Invitrogen, USA). This assay was performed in black opaque 96 well plates and the fluorescence was assessed using a microplate reader (Biotek Synergy 2 – Winooski, VT). The plate was excited at 485/20 nm, and the emitted light was measured at 528/20 nm. This reading was used to assess the change in cell numbers over time.

#### *In vitro differentiation assays of the osteoblasts on the HAp disks*

HFOb differentiation was determined by testing the cell lysates for the protein product of the bone-specific transcription factor runt-related transcription factor 2 (RUNX2), Alkaline Phosphatase (ALP), Dental Matrix Protein 1 (DMP1), and Osteopontin (OPN) activity. RUNX2 activity was measured from the lysate using a phosphospecific antibody cell-based enzyme linked immunoabsorbent assay (PACE). Specifically, 50 μL of lysate were pipetted into a protein-attachment ready microplate and diluted in PBS-T solution at a ratio of 1:1. After 24 hours the wells were endogenous peroxide quenched in 0.6% H_2_O_2_, and blocked in 10% fetal bovine serum (FBS). Anti-RUNX2 primary antibody (Cat # 41–1400, Invitrogen) was then added overnight using a concentration of 1 μg/mL, followed by PBS-T washes and a secondary antibody (Cat # 81–6720, Invitrogen) for one hour using a concentration of 0.214 μg/mL. Following 3 PBS washes, Pierce 1-step ultra TMB was added to each well and the reaction was stopped using 2 M H_2_SO_4_. The plate absorbance was measured operating the same microplate reader used in the DNA analysis. The absorbance was read at 450 nm with reference at 655 nm. Primary to secondary antibody ratio was optimized by identifying highest signal-to-noise ratio (highest signal to noise ratio found using 1ug/mL primary, 0.214 μg/mL secondary with these antibodies). ALP activity was assessed from the cell lysate using an ALP Fluorescence Detection Kit (APF, Sigma-Aldrich). 10 μL of lysate were added in black opaque 96 well plates to fluorescence assay buffer following kit manufacturer instructions. Fluorescence was read after exactly 45 minutes with an excitation of 360 nm, and an emission of 460 nm. DMP1 was detected and quantified using the same PACE technique used for the RUNX2 assay. All of the steps remained the same with the difference that the primary antibody used was anti-DMP1 (code ab76632, Abcam) at a concentration of 2.5 μg/mL, and the secondary antibody used was the same used in the RUNX2 and was used at a concentration of 0.30 μg/mL. As in RUNX2, primary to secondary antibody ratio was optimized for maximum signal-to-noise ratio (strongest signal to noise ratio found using 2.5 μg/mL primary, 0.30 μg/mL secondary with these antibodies). OPN detection from the cell lysate solution was performed using the bone panel Milliplex kit (Millipore, USA). This kit also tested for osteocalcin and osteoprotegerin. Specifically, 10 μL of lysate were used for this test and combined with assay buffer following kit manufacturer instructions.

### In vitro Collagen I semi-quantification assays of the HAp disks

A total of 10 fluorescence immunohistochemistry readings for each group were analyzed after testing semi-quantitatively for the presence of Col-I. The disks, previously fixed in 4% formaldehyde, were quenched hydrogen peroxide in 0.6% H_2_O_2_, blocked in 10% FBS, and a polyclonal anti-Col-I primary antibody (code ab34710, Abcam) was added overnight at a concentration of 10 μg/mL. This step was followed by PBS-T washes and the addition of a FITC linked secondary antibody (Code ab96895, Abcam) also using a concentration of 5 μg/mL. As in RUNX2 quantification, the strongest signal to noise ratio was found using 10 μg/mL primary, 5 μg/mL secondary with these antibodies. The disks were washed 2x in PBS-T and ProLong® Gold Antifade with DAPI was added to the bottom of the plate where the disks were inverted for fluorescent microscopy. A total of 10 intensity readings were obtained from different channels of the disks. Readings were averaged and Col-I was quantified using intensity measurements with reference to control surfaces.

### In vitro cell orientation assays of the HAp disks

**Figure 4:**
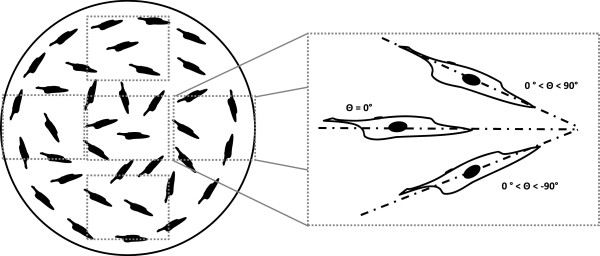
**Graphical representation of the technique used to measure the orientation of the cells growing in the flat control HAp disks.** Particular attention was paid to avoid bias orientation due to the meniscus effect due to the media on the well plate wall during cell culture. This was accomplished by measuring the cells from top to bottom and from left to right. When the long axis of the cell nuclei was parallel to the direction of the microchannels the angle was set to 0°. All the cells that were angled to the right were measured anywhere between 0 and 90°, whereas the cells angled to the left were measured with a negative number, 0 to -90°.

After staining the disks for Col-I and nuclei, the disks were analyzed under fluorescent microscope (SFL7000, Leica) for both Col-I and DAPI. Twenty random images were taken of each disk inside the artificial microchannels for both collagen and nuclei. The DAPI pictures were used to determine the angle of orientation of the cells with respect to the microchannel direction. This was done assuming that when the nucleus of the cell was elliptical in shape, the long axis of the nucleus matched the long axis of the cytoskeleton of the cell [40]. Cells undergoing mitosis were not measured for alignment. Images were analyzed using Bioquant Osteo system (Bioquant Osteo 2010, Nashville, TN). The angle of the cell was measured in degrees, with 0° being parallel to the microchannel direction, and 1 to 90° and −1 to -90° being of an angle to the right or to the left of the microchannel respectively. A schematic to describe this quantification is shown in Figure 4. When analyzing the flat control disks, bias measurement orientation due to the media-meniscus effect was prevented by taking pictures from five independent regions of interest on each sample. Successively, the blue channel images from the nuclei and the green channel from the Col-I were merged using Adobe Photoshop®.

### In vitro mechanical ECM characterization of the HAp disks

Representative disks from each investigated group were tested to determine the mechanical properties of the cell’s ECM secretions. The cells on the surface of the disks were gradually dehydrated in ethanol to preserve cellular structure. Specifically, the disks were moved into a 20/80 (vol/vol) ethanol in water for 1 hour, then 40/60 for another hour, 60/40, 80/20, and 100% ethanol two more times. The disks were then epoxied onto a 3 cm diameter metal cylinder using a very thin layer of epoxy glue. Using a commercial nano-indenter (MTS nano-indenter XP, MTS System Corporation, MN), the reduced elastic modulus (*E*_*r*_) and the microhardness (*H*) of the secreted ECM were determined. Before measuring each group the nano-indenter location was calibrated using a 5-indent template. The indenter’s microscope (20x objective) was then used to find the peaks and the valleys of the disk substrate. The difference between the channel peaks and the valleys were easily noticed at this magnification. For each experimental group the indents were made in the valleys of the microchannels. This was done because the size and the geometry of the microchannels did not allow for the positioning of the indenter tip in a normal direction to that of the surface, which has a continuously changing angle. Also, we wanted to circumvent the possibility of the nano-indenter tip coming into contact with and sliding down the curved substrate wall. The indents were performed using a Berkovich tip and a standard trapezoidal loading profile with a loading rate of 250 μN/s until a maximum load of 500 μN was applied [41]. *E*_*r*_ and *H* were determined using a previously described method [42]. Briefly, the Young’s modulus for the tested material (*E*_*m*_) was calculated by assuming that the cells had a Poisson’s ratio (*v*_*m*_) of 0.3 [41,43,44]. For the diamond indenter the Young’s modulus (*E*_*i*_) was set to 1140 GPa, with a Poisson’s ratio (*v*_*i*_) of 0.07. Equation 4 was used to calculate *E*_*m*_[42]:

(4) $$E_{m}=\frac{1-v_{m}^{2}}{\frac{1}{E_{r}}-\frac{1-v_{i}^{2}}{E_{i}}}$$

300 random readings were taken from each disk.

Hardness instead was calculated using Equation 5:

(5) $$H=\frac{P_{\max}}{A}=\frac{P_{\max}}{F\left(h_{c}\right)}$$

In which P_max_ is the peak load and A is the projected area of contact at peak load evaluated by a function which relates the cross-sectional area of the indenter to the vertical distance from its tip (*h*_*c*_) [42].

The modulus and hardness readings that fell into the control HAp readings ± standard deviation were discarded because it was assumed that the indent was measuring the actual HAp layer and not the cell ECM.

### Statistical analysis

All data is reported as average ± standard error. The statistical test used to analyze the data was a two-way analysis of variance, and Tukey’s comparison test was used to determine statistical significance between individual groups. All statistical tests were performed using SigmaPlot® (version 11.0, Systat Software, Inc.). Differences were considered significant at P < 0.05. The difference between variances of distribution was evaluated using an F-Test (MedCalc® V 12.2.1.0).

## Results

### Characterization of the Hap disks

**Figure 5:**
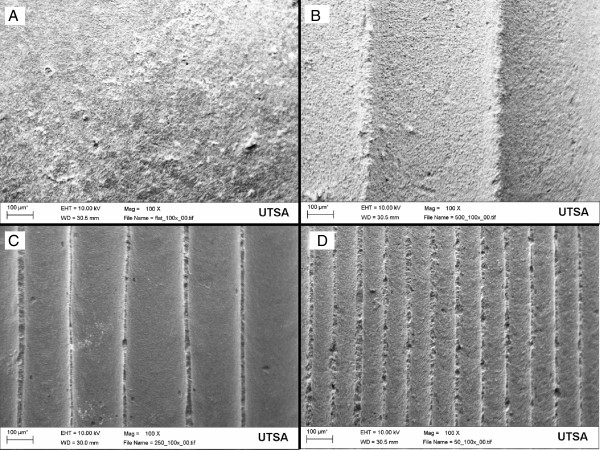
**Scanning Electron Microscopy (SEM) pictures of the microchannel templates in each of the three dimensions fabricated: the flat control substrate (A), the 500 μm (B), the 250 μm (C) and the 100 μm template (D).** Magnification is 100x, and the scale bar length is 100 μm.

Reproducible disks with longitudinal curved microchannels spanning the entire disk were created using the casting technique. This novel geometry was analyzed under SEM and constant microchannel diameter was seen throughout the four disk groups (Figure 5). Constant crystal size was previously determined with our slurry HAp technique [39]. The measurements for *λ*, *a*, and *l* are reported in Table 1. The unsintered template microchannels measured 120, 320, and 750 μm in diameter, and after the sintering process HAp underwent compaction due to binder loss that resulted in channel sizes of 100, 250 and 500 μm in diameter, the same *λ* dimensions that match the physiological size range of naturally occurring osteons [30]. For group identification these three groups will be labeled as 100 μm microchannel diameter (100mc), 250mc and 500mc.

**Table 1:** Results of morphology characterization of the HAp disks

**Template (μm)**	**λ (μm)**	**a (μm)**	**l (μm)**	**Area (mm**^**2**^**)**
100	92.32 ± 0.57	42.83 ± 0.92	169.55 ± 2.12	79.78 ± 0.87
250	229.92 ± 1.07	111.84 ± 2.24	430.27 ± 5.41	81.25 ± 0.92
500	525.07 ± 2.32	203.18 ± 3.40	880.27 ± 9.00	72.78 ± 0.51

All measurements were taken after thin histological longitudinal sections of the disks were cut, polished, and analyzed using Bioquant Osteo®. All values reported with standard error of the mean (SEM).

### HFOb growth and differentiation in the microchannels

**Figure 6:**
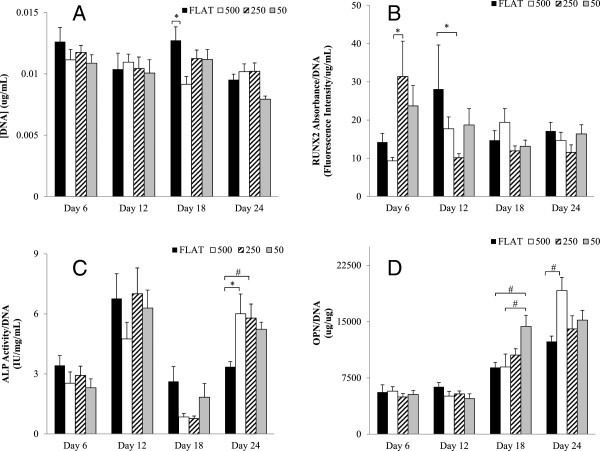
**HFOb cell count and differentiation assay for the four groups tested. (A)** cell count for the Flat, 500, 250 and 100 mc throughout four time points: 6, 12, 18, and 24 days. No proliferation is seen throughout the four weeks. There is a significant decrease in cells found on the 100 group at the last time point. Also seen are the differentiation markers RUNX2 **(B)**, ALP **(C)**, OPN **(D)**. The RUNX2 groups do not show any significant difference between the groups although we can see a trend in which the 250 μm diameter microchannels have lower levels than the rest of the groups. The ALP activity shows two different peaks at day 12 and at day 24. At the last time point the 500 and 250 μm diameter microchannels showed a significant higher ALP activity than the Flat control group. The OPN levels do show significant difference on day 18 between the 100 μm diameter microchannels and the control group. At day 24 the significant difference was seen between the 500 μm and the Flat. All data shown as mean ± standard error. * P < 0.05; ^ P < 0.01; ^#^ P < 0.001.

The dsDNA assay demonstrated that throughout the 24 day experiment the cells did not show significant changes in cell number. This is consistent with a mature osteoblast phenotype. This is likely to have occurred because all the cells that were seeded in the disks were signaled to differentiate by the dexamethasone in the osteogenic media. These results are shown in the chart in Figure 6A.

**Figure 7:**
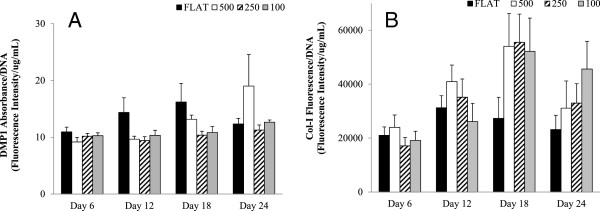
**Chart showing HFOb differentiation marker DMP1 (A) and Collagen-I fluorescent assay (B). (A)** shows no significance between any of the groups or time points, probably due to the fact that this marker is very late for differentiation and we saw no osteoblasts becoming osteocytes. **(B)** shows Col-I fluorescent assay results that are normalized to DNA. The results show a trend in which the flat control group has less collagen than the rest of the groups. There is no significant difference between the groups at each time point. Other markers tested included osteocalcin and osteoprotegering, but levels detectable levels were too low. All data shown as mean ± standard error.

The four differentiation markers analyzed were chosen for being indicative of early through late stage osteoblast differentiation. Of the four markers, RUNX2 is activated at the earliest time [45], Figure 6B. RUNX2 results showed the highest levels at day 6 and then decreased through the remainder of the experiment. The earlier time point shows that the 250mc and the 100mc have the highest trend of RUNX2 expression. By day 12 the 250mc decreased to its lowest level while the control group spiked, demonstrating delayed differentiation. The second chronological differentiation marker is ALP, Figure 6C. The data supports this by showing an ALP spike on day 12, and on day 24 [46]. At this time point, the 500mc and the 250mc were significantly different from the flat control group (P < 0.001 and P < 0.05 respectively). OPN is chronologically an even later differentiation marker of osteoblasts, and the results are shown in Figure 6D. This data also correlates with our findings and after being minimal on days 6 and 12, they rise on day 18 to have the highest spike at day 24. In particular, the 100mc had a significantly higher amount of OPN than the flat control group on day 18 (P < 0.05) while the 500mc showed a significantly higher level than flat control on day 25 (P < 0.05). The last differentiation marker tested was DMP1. These results can be seen in Figure 7A. This marker tests for osteoblast differentiation into osteocytes. The data for this marker shows very slight to no change in time in the expression of this marker. Although possible spikes of DMP1 are seen in the control flat disks at days 12 and 18, and for the 500mc at day 24, none of these changes are significantly different.

### In vitro extracellular matrix (ECM) assays of the HAp disks

**Figure 8:**
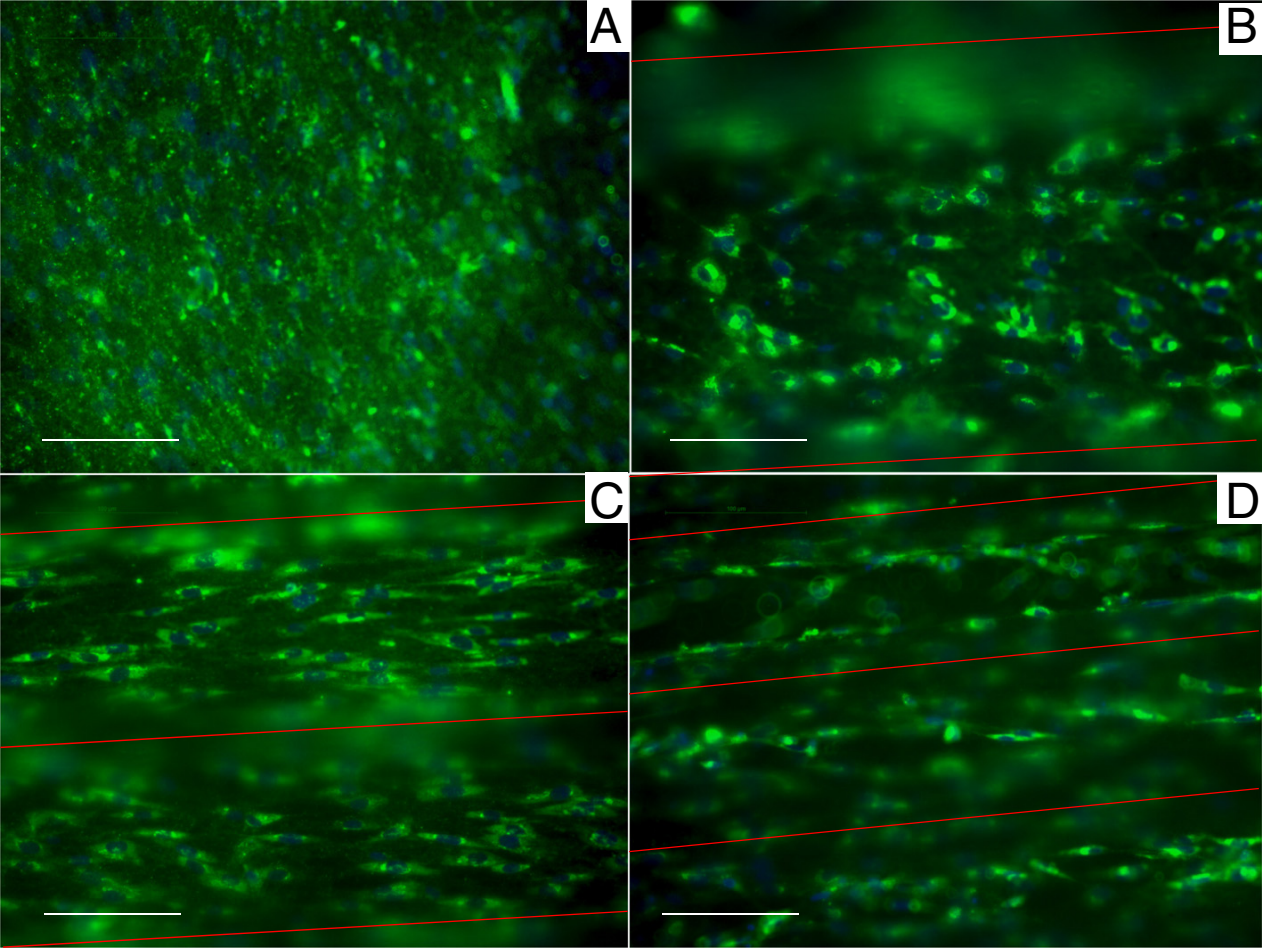
**Fluorescent photographs of Col**-**I stain at day 6 in the experimental groups.** The flat control **(A)** does not show any type of organization. The 500mc group **(B)** shows very early signs of organization even though the individual cells have not completely chosen direction. The 250mc group **(C)** shows cellular distribution which is allot more organized than the previous groups. Last, the 100mc group **(D)** shows early signs of differentiation already at day 6. Scale bar corresponds to 100 μm. In each figure, the red lines indicate the direction of the microchannels as compared to the cell animation.

A total of 10 readings were analyzed in the quantification of Col-I fluorescence in each group. These findings are shown in Figure 7B. Although the data was not significantly different, a trend is seen in which all of the tested groups from day 18 produced greater Col-I than the flat control group. The highest values of Col-I were seen at day 18 and slowly decreased by day 24 to roughly the same levels as day 12. Figure 8 shows collagen staining within the different size microchannels.

### In vitro cell organization and orientation assays of the HAp disks

**Figure 9:**
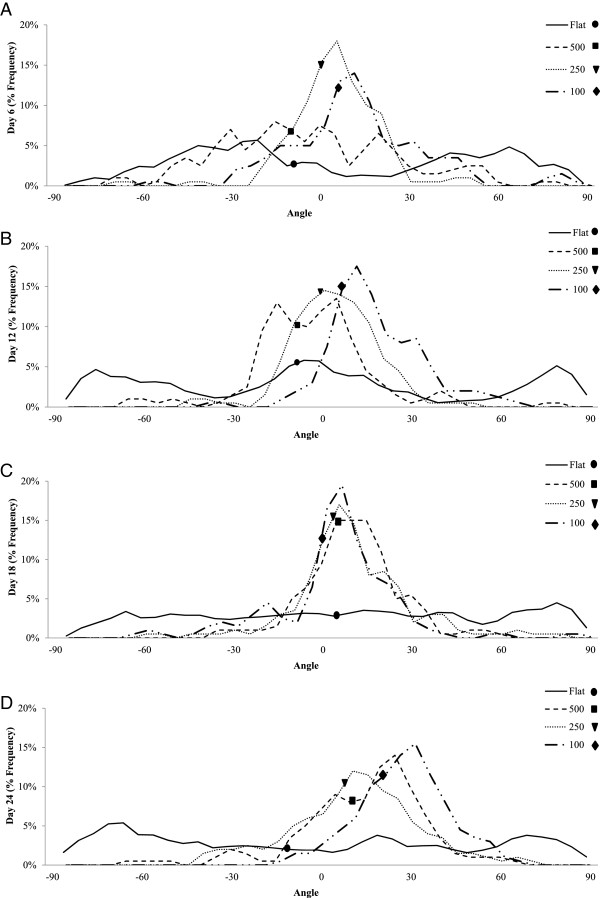
**Frequency distribution of the cell angles during the four time points.** The dots represent the median angle of orientation for each group. This graph shows the early stages of cell organization following cell attachment and early differentiation. In the control flat group the cells appear to be very disorganized and are scattered in every position. Overall all of the experimental groups showed higher organized than the flat control groups which remained disorganized for the entire 24 days (significantly higher variance^#^). At day 6 **(A)** the 250mc group shows earlier orientation than the rest of the groups^#^, followed by the 100mc group. The 500mc group does not quite achieve early organization. By day 12 **(B)** the 100mc group gains the most organization* followed by the 250mc and the 500mc group. The 500mc group reaches organization by this time. At day 18 **(C)** the most organized groups remain the 100mc and the 250mc^. The 500mc becomes even more organized as well. At the very last time point **(D)** we see the 100 group that shows higher organization than the rest of the groups (P < 0.001), followed by the 250mc and 500mc which show no difference from one another.* P < 0.05; ^ P < 0.01; ^#^ P < 0.001.

The cell organization and orientation within the microchannels was determined from computational analysis of DAPI-stained cell nuclei. Although the same data was used, cell organization was analyzed comparing the change in orientation between the different mc diameters at each time point, whereas cell orientation was studied by comparing the orientation of each individual investigational group through time. The results showed that the cells growing in the flat control group did not become organized in 24 days. Figure 9 shows the measured frequency distribution of cellular orientation. Narrow frequency curves were characterized by lower standard deviation, and translated to higher cellular organization on the substrate. Correspondingly, wider frequency curves were represented by a higher standard deviation, indicating low organization of the cells. At day 6 (Figure 9A), the variances of all the samples were statistically significant from one another (P < 0.01), with the 250mc having the lowest variance followed by the 100mc and the unorganized 500mc. At day 12 (Figure 9B) the 100mc displays statistically higher organization than the rest of the groups (P < 0.05), followed by the 250mc and the 500mc. The 500mc in fact shows early organization trends that differ from the control (P < 0.001). At day 18 (Figure 9C) the most organized groups remain the 100mc and the 250mc (P < 0.01). The 500mc continues to become more organized as well. By day 24 (Figure 9D) the 100mc once again shows higher organization than the rest of the groups (P < 0.001), followed by the 250mc and 500mc which show no difference from one another. The change in cellular orientation was also evaluated within each group over time from day 6 to day 24. The control group was never able to gain orientation, and therefore the median angle was never analyzed. However the 500mc showed a significant change in median angle orientation with time (P < 0.05) from −11.30°, to −7.05°, to 5.40° and finally 11.56°. The 250mc displayed a significant change in orientation from day 6 to day 24 (P < 0.001) starting from −0.89° and ending at 8.16°, with the most significant changes happening between days 12 (−1.69°) and 18 (2.69°) (P < 0.001). The 100mc showed a very significant change between day 6 and 24, going from 4.43° to 21.88° respectively (P < 0.05), exhibiting perhaps the largest shift in cell orientation amongst the groups. The 250mc remained stable and close to the 10° angle. Overall, all the microchannel groups were more organized than the control group which remained disorganized for the entire 24 days.

### In vitro mechanical ECM characterization of the HAp disks

**Figure 10:**
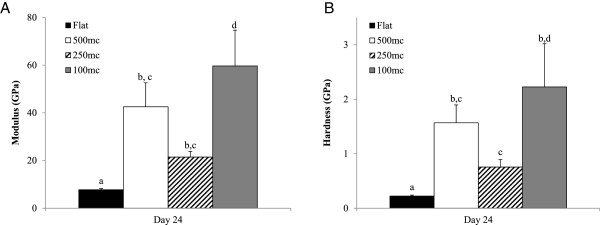
**The elastic modulus (A) and the hardness (B) of the secreted ECM at day 24 across different substrates are shown.** In both graphs all of the experimental groups were significantly different from the control flat group. The 100mc group had the highest modulus value than the other groups, and also had a higher hardness value than the 500mc group. Thus it can be concluded that the curvature had an effect on ECM secretion and mineralization in the 100mc group that is not seen in the other flat substrates. Letters show statistical significance between groups (p < 0.05), where a,b,c,d are used to indicate statistical differences between groups where any two groups unconnected by a common letter are significantly different (p < 0.05).

Elastic modulus and hardness of the secreted ECM was analyzed using a nano-indentation technique. The experimental samples were compared to pure HAp substrate to determine when the indenter was in contact with cell ECM or in contact with the pure HAp substrate. The results from the experimental groups were all significantly different from the control HAp substrate for both the modulus and the hardness (P < 0.001). When analyzing the elastic modulus of the secreted ECM (Figure 10A), the 100mc had the highest value, and was significantly different from all other groups (P < 0.05). Even though the 500mc and the 250mc were not significantly different, a trend was observed in which the 500mc had higher modulus than the 250mc group. All of the experimental groups had higher moduli than the flat control group (P < 0.05). The same trend was seen in the hardness values (Figure 10B), with all microchannel groups having significantly greater hardness than the flat HAp disk (P < 0.05).

## Discussion

Multiple materials, surface morphologies, and cell types have been previously investigated to identify the role of surface architecture on tissue regeneration. In fact, micropatterned surfaces have been previously created on different material substrates using a variety of techniques. Primarily these methods were micromachining [47-49], low voltage electron beam lithography [34], standard photolithographic [50-52] and photopolymerization process [53], plasma oxidation [33,54], and single mask fabrication technique [35]. All these techniques can achieve a different array of substrates, but none of them was useful to create the microchannel template on HAp. Other materials used to identify the effects of substrate on cell behavior include silicon wafers [33-35,48], polystyrene [36,52], methacrylate [53], Perspex [51], SU-8 5 photoresist [50], latex [36], and ligand patches [37]. HAp posed as a more challenging material to machine and/or pattern due to the strength and brittleness of its nature. Multiple cell types including tenocytes [50], fibroblasts [32,35,37,48], myoblasts [33,55], neurons [51], spiral ganglion cells [22], retinal endothelial cells [56], epithelial cells [9], astroglial cells [52], HeLa S3 cancerous cell line [36], and Mouse B16F1 melanoma cells [36] have been previously investigated regarding their response to surface architectural cues. However, there have been very few (or no known reports to the authors knowledge) reports on the response of fetal osteoblasts on such curved or micro-patterned substrates. In this study, a mold template/casting technique for HAp was employed. This allowed for precise, repeatable (<5% variation), and consistent (< 2.5% variation) patterned morphologies resembling the natural range of osteon curvature (Table 1). Using this system, we systematically studied the effect of a changing substrate curvature within the range of native osteon diameter on osteoblast response while simulating lamellar organization.

Osteoblast precursor cells such as the ones used in this study are not fully differentiated bone cells, however they are already committed to the osteogenic lineage. These cells undergo three key phases of cell activity: proliferation, differentiation and mineralization [57]. These steps are well defined and can overlap one another [58]. The osteoblast precursor cells seeded on the disks were induced to differentiate into mature osteoblasts using the glucocorticoid dexamethasone. A common drawback of this steroid is a reduction in the rate of replication of the cells [59]. Thus, as osteoblast precursor cells start to differentiate, proliferation ceases. This is demonstrated with the findings on DNA analysis in the current study which showed no significant change in cell number but rather only differentiation. The assay used to determine cell numbers relies on testing from cell lysate solution. Given both the geometry of the substrate and the time for which the cells were cultured, it is not surprising that the DNA yield was not optimal, translating into high standard error. Another drawback related to use of Dexamethasone is the inhibition of bone formation in vivo due to decreased collagen synthesis [59]. Overall in this experiment the dexamethasone ρρdid not inhibit collagen secretion altogether, since actual Col-I deposition was seen (Figure 8). Our results show a strong differentiation response consistent with the effect of dexamethasone, glucocorticoid receptor induced activation of osteoblast gene expression such as osteocalcin, collagen Iα1 and transforming growth factor-β1. Osteoblasts differentiation can be detected by analyzing specific early and late markers which have been thoroughly studied and characterized [60]. Perhaps the earliest activation factor is RUNX2, followed by ALP (an early-mid marker), OPN, OC, ON [61]. The four week experiment resulted in no changes in osteoblast differentiation/mineralization rates between the four experimental groups, concluding that the curvature associated with different microchannel diameters had little or no effect on HFOb rate of differentiation. The rate of differentiation for all groups in this study was consistent with the literature. RUNX2 peaked early at week 1, followed by ALP, which was activated first at day 12 and then at day 24. OPN, which is activated by RUNX2 [62], did not show activation until day 18, with the highest levels seen at day 24, showing continuous differentiation throughout the study. The expression of DMP1 was limited after 24 days, which does not agree with the study by Mikami *et al.* which showed early expression of DMP1 from mRNA using RT-PCR when using dexamethasone as stimulant [63]. However, the current study did not analyze DMP1 expression from mRNA but rather quantified the actual Human DMP1 protein present in the cell lysate at the different time points. It is difficult to determine whether natural DMP1 expression follows the same *in vitro* trend. Narayanan *et al.* gave an insight into the role DMP1 [64]. According to their study in the very early stages of differentiation, DMP1 serves as a transcription factor that stimulates further differentiation and expression of such markers as RUNX2. Only at the last stage of Ob maturity (near collagen mineralization) is DMP1 secreted by the differentiating osteoblast and triggers mineralization [64]. This contradicts the findings that showed different modulus and hardness of the deposited ECM, suggesting that mineralization is occurring in the curved substrates, whereas there were no differences in the flat substrate. According to the findings in the current study, a noticeable increase in DMP1 should have been expected at four weeks. These contradicting discoveries could be due to the deterioration of the DMP1 after freeze/thaw cycles, or due to the low sensitivity of the DMP1 ELISA. Thus, it is reasonable to conclude from the present study that negligible increases in DMP1 expression observed from day 6 to 24 in all tested groups indicated the absence of phenotypic transition from early osteoblast to late mineralization behavior. DMP1 is also a marker for osteoblast differentiation into osteocyte. The lack of DMP1 could be explained by the cells not reaching mature osteoblast status at 24 days, or by the inhibited proliferation of the cells in culture, preventing layering effects in the microchannels.

When Luan *et al.* tested osteoblast cultures for Col-I secretion at 4, 8, and 12 days they were unable to see any significant differences at each time point [65]. Refitt *et al.* instead investigated the function of silicone in Col-I secretion. The Col-I measurements they described were done indirectly on the amount of carboxy-terminal propeptide of type 1 procollagen liberated into the culture medium [66]. In this study, although a definite trend can be seen in collagen secretion up to day 18 in all tested groups, no significant differences in collagen deposition over time or between groups were seen. However, a change of trend in the Col-I fluorescence was observed at day 24 and was characterized by a decrease in fluorescence intensity throughout all four experimental groups. This change, although not significant, was attributed to early mineralization that contributed to the masking of the Col-I antibody labeling. The control group did not show any changes in Col-I secretion within the study, which is consistent with data reported by Luan *et al.*[65].

Previous studies on cell orientation within micropatterned substrata all agreed that the narrower the channel, the higher the degree of alignment the cell presents, and the deeper the groove, also the higher the degree of alignment [49-53,55]. There are however a few basic differences between the cited research and those performed in the current study. The micropatterning described in the literature is in the range of few micrometers and does not necessarily reflect the concave shape that this study created. In fact Recknor *et al.* and Kapoor *et al.* had squared grooves [50,52], Brunette *et al.* had both vertical and sloped walls [49], and Charest *et al.* had 10 μm holes and 10 μm grooves [55]. Another important difference is that most studies only lasted up to 3 days and the cells were seeded at minimal confluence with the purpose of analyzing individual cell’s behavior. The present study, however, lasted 24 days, and the disks were seeded at full confluence with the goal of analyzing osteoblast behavior as a tissue, observing its mineralization and tissue orientation in relation to the substrate. The behavior of the individual cells was not as imperative. This experiment was able to show overall organization in the 100 μm, 250 μm and 500 μm, as well as the change in orientation with time which may be part of the signaling mechanism for the change in lamellar alignment observed in natural alternating osteons. Although initial cell layering was observed in the channel valleys from the beginning, it was not shown to change density over 24 days. A previous study by Kacena et al. showed that gravity plays no effect on fully confluent osteoblast that are not in proliferation mode [67]. No effect of gravity or cell slippage within the microchannels was observed in the current study either.

The nanomechanical analysis demonstrated that by day 24 the ECM modulus and hardness values were much higher in the 100mc than the flat substrate and other microchannel groups. It is hypothesized that the high modulus and hardness values seen were due to the process of mineralization of the ECM by day 24, a process that perhaps was not seen in the flat substrates. The cell line investigated lacked phenotypic transition from early osteoblast to one that has late mineralization behavior. This can be explained by the cells not reaching mature osteoblast status at 24 days, or by the inhibited proliferation of the cells in culture, preventing layering effects in the microchannels.

It can be speculated that the curvature of the substrate indirectly increased the rate of mineralization of the HFObs on the ECM. This mineralization trend was also supported by the ALP activity and the OPN levels, which were significantly higher in the 100mc group than the flat control group. When visually comparing the organization of the cells and their change in orientation with the modulus and the hardness, it was observed that the higher the organization of the cells in the substrate, the higher the modulus and the hardness. As observed above, at day 24 the 100mc displayed the highest cell organization, followed by the 500mc and the 250mc. On the other hand, the flat substrate never demonstrated orientation. As the cells aligned onto the substrate, they were able to organize faster and secrete organized ECM with higher modulus and hardness. After 24 days in cell culture, cells produced their own extracellular matrix that could modify the topography of the substrates. In fact, the rate of change on each of the microchannels groups is probably different since the cells are probably laying down ECM to vary their local micro-environment. This could explain the different temporal response elicited on each microchannel. During nano-indentation testing we measured E and h of the plain HAp disks to determine baseline values (E = 125.30 ± 9.52 and h = 8.98 ± 1.25). This value was considered the internal control to verify that all the indents on the in vitro cultured substrates were measuring actual secreted ECM properties and not the underlying HAp surfaces. Also, HAp’s E was 2 fold higher than the 100mc group and 6 fold higher than the control flat groups. The hardness was 4 fold higher than the 100mc group and 9 fold higher than the Flat disk control. The elastic modulus value of the flat substrate (7.8GPa) was slightly lower than what has been previously reported in the literature for dehydrated human bone (12.4GPa) [41,43,68-70]. However, the three experimental groups with different diameter microchannels resulted in a much higher modulus. Specifically, the 100 μm group showed the highest modulus amongst the groups (42.6GPa) and tested above human bone (13.4-25.7GPa) [41]. The hardness values of the flat control disk (0.2GPa) averaged below previously reported values for human bone (0.47-0.74GPa) [41,43,68-70]. The experimental groups with microchannels have much higher hardness than what was previously reported, and once again, the hardness in the 100mc (1.6GPa) is above natural bone values. It is hypothesized that the reason this group tested above cortical bone is because the experimental setup in this study allowed us to measure modulus and hardness directly on the surface of the osteon. When testing natural bone, the values come from an area outside the osteon, and this could contribute to the differences seen here. Potentially due to a largely two dimensional environment, in this study there were no significant changes in differentiation markers, although a significant difference in matrix modulus and hardness was observed, indicating different collagen expression and mineralization between groups occurred. This is something that should be further investigated in future studies, especially in a 3D environment.

The goal of this study was to determine osteoblast response to being cultured on a curved substrate mimicking the native curvature of an osteon. This was investigated through the alignment of cells in the microchannels (organization), the change in orientation with time, the secretion of collagen, and the production of a mineralized extracellular matrix. In this study we showed that the substrate affects physical properties of cellular organization and orientation, as well as the composition and quality of the ECM secreted. Regardless of what substrate the cells were cultured in, they all maintained equal number of cells over time while differentiating at statistical equal rates. Although this model is only the first step in depicting osteoblast behavior within a three dimensional osteon, it is critical since it has allowed us to demonstrate that when cultured in the appropriate substrate curvature and appropriate conditions, osteoblasts can organize as a tissue and change orientation with time, producing a mineralized ECM that is hard and tough. Based on these findings, future studies will focus on recreating three-dimensional scaffolds with longitudinal microchannels that resemble naturally occurring osteons. The scaffolds will then be characterized for their ability to recreate an *in vitro* osteon.

## Conclusion

Overall, in this study microchannels of 100, 250 and 500 μm diameter were successfully created on the surface of HAp disk. A 24 day *in vitro* study was performed on these different substrates and demonstrated an early osteogenic phenotype and extensive collagen deposition Moreover, cell alignment within the microchannels were assessed to find that the 100mc and the 250mc groups induced fast orientation and a higher degree of organization, while the 500mc only started showing organization after 12 days and the control flat group did not show alignment towards a specific direction. This study was also able to demonstrate a significant increase in elastic modulus and hardness in the microchannel substrates compared to a flat control, suggesting that curvature plays an important role in the quality of the ECM secreted and mineralized. Understanding how bone cells grow and secrete ECM in these curved substrates is the first step in understanding the mechanism involved in creating artificial osteons and in the long run regenerating organized cortical bone.

## Competing interests

The authors declare that they have no competing interests.

## Authors’ contributions

Study design: MP, MA, SS and TG. Study conduct: MP. Data collection: MP and SS. Data analysis: MP and TG. Data interpretation: MP and MA. Manuscript preparation: MP, TG, MA and SS. Approving final version of manuscript: MP, MA, TG and SS. MP takes responsibility for the integrity of the data analysis. All authors read and approved the final manuscript.
